# Associations Among Methylene Tetrahydrofolate Reductase rs1801133 *C677T* Gene Variant, Food Groups, and Non-alcoholic Fatty Liver Disease Risk in the Chinese Population

**DOI:** 10.3389/fgene.2021.568398

**Published:** 2021-02-18

**Authors:** Xiaoyan Hao, Cong Ma, Tianyuan Xiang, Lei Ou, Qiang Zeng

**Affiliations:** Health Management Institute, The Second Medical Center & National Clinical Research Center for Geriatric Diseases, Chinese PLA General Hospital, Beijing, China

**Keywords:** food groups, non-alcoholic fatty liver disease, methylene tetrahydrofolate reductase, risk factors, lifestyle, homocysteine

## Abstract

**Objectives:**

To investigate the associations among the methylene tetrahydrofolate reductase rs1801133 *C677T* gene variant, food groups, and the risk of non-alcoholic fatty liver disease in the Chinese population.

**Methods:**

A study of gene polymorphism was conducted using the polymerase chain reaction method. A total of 4,049 adults participated in the study, and all underwent physical examination and genotyping. Participants filled out a dietary questionnaire to enable us to assess the frequency and quantity of food consumption.

**Results:**

The important variables identified as risk factors of non-alcoholic fatty liver disease were age, smoking, sex, body mass index, hyperlipidemia, diabetes, and methylene tetrahydrofolate reductase genotype (T – allele carriers). The homocysteine content was higher in the non-alcoholic fatty liver disease group than in the control group, and was higher in the T- allele than C- allele carriers. The homocysteine content was the highest in the T- allele carriers. Additionally, certain food groups such as milk and beans were associated with a lower risk of non-alcoholic fatty liver disease. Food groups such as meat, were associated with a higher risk of non-alcoholic fatty liver disease. Fresh fruit and vegetables, salted and smoked foods, desserts, cereals, fish, and eggs were not associated with the risk of non-alcoholic fatty liver disease. However, the influence of salted and smoked foods on non-alcoholic fatty liver disease was different in the C-allele and T-allele carriers of methylene tetrahydrofolate reductase (CT + TT vs. CC, OR = 1.196, *P* = 0.041 for 1–4 times food per week, OR = 1.580, *P* = 0.004 for 5–7 times per week). Similarly, salted and smoked foods were also a risk factor for the development of non-alcoholic steatohepatitis in patients with non-alcoholic fatty liver disease.

**Conclusion:**

This study found that the T-allele of the *C677T* variant of methylene tetrahydrofolate reductase was a risk factor for non-alcoholic fatty liver disease among Chinese people. These results can likely aid the development of novel approaches for managing non-alcoholic fatty liver disease risk.

## Introduction

The prevalence of non-alcoholic fatty liver disease (NAFLD) is increasing worldwide (Younossi et al., 2016; Subada et al., 2018). NAFLD is a disease caused by excessive fat in the liver (Yki-Järvinen, 2014). According to this report, NAFLD is now an epidemic in China, and the prevalence of NAFLD in South China is 45.4% (Yi et al., 2017). Therefore, identification of modifiable risk factors for the prevention of NAFLD is urgently needed for Chinese patients. NAFLD is a multiple factor disease that is influenced by genetic variation, diet, age, sex, ethnicity, and lifestyle, along with conditions including obesity, insulin resistance, dyslipidemia, hypertension, diabetes mellitus, and metabolic syndrome (Hamabe et al., 2011; Satapathy and Sanyal, 2015; Kalia and Gaglio, 2016; Tong and Guo, 2019; Zhang et al., 2019). Several studies have been conducted on the association between gene polymorphism and NAFLD (Kong et al., 2017; Liu et al., 2019; Xu et al., 2019). Specifically, some studies have indicated that susceptibility genes may play an important role in the pathogenesis of NAFLD (Sookoian and Pirola, 2011; Yang et al., 2011). Methylene tetrahydrofolate reductase (MTHFR) converts 5, 10-methylenetetrahydrofolate to 5-methyltetrahydrofolate (Froese et al., 2016). The human *MTHFR* gene is mapped to chromosome 1p36.3 (Rosenberg et al., 2002). Moreover, the *MTHFR rs1801133 C677T* gene variant is associated with enzyme activity (Wan et al., 2018). It is known that *MTHFR* variation will result in a reduction of MTHFR enzyme activity. Specifically, the substitution at 677 bp of the *MTHFR* gene is a common mutation that leads to hyperhomocysteinemia (Liew and Gupta, 2015). *MTHFR C677T* has also been associated with NAFLD (Adinolfi et al., 2005). Furthermore, the lack of choline can lead to hyperhomocysteinemia and NAFLD (Leclercq et al., 2000; Liu et al., 2014), and the *MTHFR rs1801133 C677T gene variant* has been associated with choline status (Abratte et al., 2008). Therefore, homocysteine content can indirectly reflect the choline content. However, choline status is also related to daily diet, and different food groups have varying choline content. Foods that contain choline include liver, beef, fish, pork, fruits, and vegetables, milk, beans, desserts, and salted and smoked foods (Wiedeman et al., 2018; Probst et al., 2019; Ullah et al., 2019). Therefore, the influencing factors between different groups may be different, depending on the interaction of genetic variation and lifestyles (Ma et al., 2017). Although there is growing evidence of a strong relationship between *MTHFR* variation and NAFLD (Sazci et al., 2008; Franco Brochado et al., 2013; Catalano et al., 2014), the relationship between food groups and NAFLD in different groups has rarely been studied. Here, we systematically analyzed the associations between *MTHFR rs1801133 C677T* gene variant and food groups in relation to the risk of NAFLD in the Chinese population. We hope that these results will provide novel ideas for the management of NAFLD risk.

## Materials and Methods

### Subjects

The data were collected from July 2018 to July 2019 at the Health Management Institute of Chinese PLA General Hospital. The studies involving human participants were reviewed and approved by the Institutional Ethics Committee of Chinese PLA General Hospital. All the methods were performed in accordance with relevant guidelines and regulations. The study was conducted in accordance with the World Medical Association’s Declaration of Helsinki, and all participants provided written informed consent prior to the study. According to the standardized criteria, fatty liver was diagnosed by abdominal ultrasonography (Saadeh et al., 2002; Hashimoto et al., 2013). All subjects underwent ultrasonography by the same experienced radiologist and using the same equipment. NAFLD was diagnosed according to relevant guidelines and regulations (Fan et al., 2019). The normal control individuals were selected based on abdominal ultrasonography, but those with liver disease were excluded. Finally, a total of 4,091 adults participated in this study, and all underwent both physical examination and genotyping. Among these, 42 patients were excluded for the following reasons: 10 did not sign an informed consent form, 20 did not provide a completed questionnaire, and 12 had inadequate blood samples. Therefore, 4,049 participants were included in this study. Liver fibrosis is the main cause of mortality in patients with non-alcoholic steatohepatitis (NASH), and the BARD score was used to recognize patients that are at high risk of developing advanced fibrosis (Harrison et al., 2008). On the basis of this score, we further screened patients with liver fibrosis from among those with NAFLD. Importantly, patients with a positive BARD score (>2 points) were considered to have liver fibrosis.

### Detection of the *MTHFR* Genotype and Biochemical Indicators

*MTHFR* polymorphisms were detected by using a gene chip hybrid analysis. Venous blood samples were collected in ethylenediaminetetraacetic acid (EDTA) tubes. DNA was extracted from whole blood using the QIAamp^*R*^ DNA Mini Kit (CAT No. 51304, Germany). The *MTHFR* genotype was determined by PCR-genotyping microarray analysis of three gene types (MTHFR Genotyping kit, BaiO, Shanghai, China). The detection of *MTHFR* mutation was performed in accordance with the manuals of the Gene Array Kit (BaiO Genotype Detection Gene Array Kit, Cat. No. 51304, Germany) and equipment (BaiO Technology Corp., Shanghai, China). Homocysteine (Hcy) levels were determined by fluorescence detection (F-1080, Hitachi Ltd., Tokyo, Japan) on high-performance liquid chromatography (HPLC; LC-9A, Shimadzu Corp., Kyoto, Japan). Fasting plasma glucose (Glu) was detected by using the hexokinase method. Last, the total cholesterol (TC), high-density lipoprotein (HDL), triglyceride (TG), hemoglobin (Hb), alanine aminotransferase (ALT), and aspartate transaminase (AST) were measured by an autoanalyzer (Cobas c 501 autoanalyzer, Roche Diagnostics, Germany).

### Evaluation of Food Groups

As recommended by the China Health and Nutrition Survey (CHNS) (Ge, 1995) and our slightly changed forms (Ma et al., 2017; Zeng et al., 2017) based on the current Chinese lifestyle, all subjects were asked to provide food-related information through a self-questionnaire at their first visit to enable us to assess their diet. The questionnaire included items on socio demographic characteristics, smoking, eating habits, drinking, family history, and medical history, including drug use, allergies, and surgeries. The food groups included cereals, fish, eggs, fruits, and vegetables, soy products, salted, and smoked foods, desserts, milk, and meat. The food frequencies were ranked from 1 to 3 (1: less than 1 day per week. 2: 1–4 days/week. 3: 5–7 days/week). The daily quantity of food groups consumed was estimated as follows: cereal (100–500 g/day), meat (100–200 g/day), fruits and vegetables (200–500 g/day), sugar (30–50 g/day), and salt (6–8 g/day). The dietary intake collection method that was used has been formally validated in the Chinese population (Ma et al., 2017; Zeng et al., 2017), and other questionnaires used the estimated diet record, which is reliable and widely accepted (Bonifacj et al., 1997).

### Statistical Analysis

Student’s *t*-test, one way-ANOVA, chi-squared tests, Fisher’s exact test, and binary logistic regression were performed using SPSS version 24.0. A Hardy–Weinberg equilibrium analysis was performed by using the chi-square test. All analyses were performed with 95% confidence intervals, and *p*-values < 0.05 were considered to indicate statistical significance.

## Results

### Hardy-Weinberg Equilibrium

The results showed that the genotypes of the NAFLD patients and controls conformed to the Hardy-Weinberg equilibrium. Table 1 shows the genotype and allele frequencies in NAFLD and controls.

**TABLE 1:** Hardy–Weinberg equilibrium testing for MTHFR C677T genotypes in the NAFLD and control groups.

Genotype	NAFLD	HWE *P*-value	Control	HWE *P*-value
CC	584	*P* = 0.856	355	*P* = 0.822
CT	1236		694	
TT	782		398	

**P*-value > 0.05 indicates that the studied population were in HWE.*

### Clinical Characteristics

In total, 4,049 patients were included in the study. The frequencies of *MTHFR* alleles were 23.19% (CC), 47.67% (CT), and 29.14% (TT). The average age of patients with NAFLD was 53.82 ± 7.46 years. The important variables identified as risk factors of NAFLD were age, sex, BMI, smoking, hyperlipidemia, diabetes, and *MTHFR* (T allele carriers) (Table 2). Importantly, subjects carrying CT or TT were at a higher risk of developing NAFLD than those carrying CC (OR = 1.233, 95% CI: 1.010–1.506, *P* = 0.04). Waistline and hypertension were not risk factors for NAFLD (*P* > 0.05). However, factors such as age, waistline, BMI, smoking, hyperlipidemia, hypertension, and diabetes were not significantly different between the C- allele and T- allele carriers (Table 3).

**TABLE 2:** Demographic clinical characteristics in the NAFLD and control groups.

Characteristics	NAFLD (2602)	Control (1447)	*P*-value	Odds Ratio (95% Confidence Interval)
Age	53.82 ± 7.46	51.65 ± 8.61	<0.001	1.03 (1.018–1.041)
Sex	2149 (82.59)	672 (46.44)	<0.001	2.234 (1.807–2.761)
BMI	26.91 ± 2.89	22.90 ± 2.47	<0.001	1.712 (1.63–1.798)
Waistline	93.49 ± 8.74	81.7 ± 22.73	0.199	1.008 (0.996–1.021)
Smoking	970 (37.28)	290 (20.04)	0.001	1.413 (1.149–1.737)
Hypertension	417 (16.03)	76 (5.25)	0.339	
Hyperlipidemia	1693 (65.07)	620 (42.85)	<0.001	1.517 (1.277–1.803)
Diabetes	207 (7.96)	26 (1.79)	0.004	2.016 (1.248–3.256)
MTHFR (T allele carriers)	2018 (77.6)	1092 (75.4)	0.04	1.233 (1.010–1.506)

*Continuous variables were expressed as means ± standard deviations when normally distributed, and categorical variables were expressed as percentages (*n* = 4,049).*

**TABLE 3:** Demographic clinical characteristics in the C- and T- allele carriers.

Characteristics	CC(939)	CT + TT(3110)	*P*-value
Age	52.98 ± 8.15	53.06 ± 7.89	0.243
Sex	669 (71.25)	2152 (69.19)	0.014
BMI	25.49 ± 3.48	25.47 ± 3.31	0.298
Waistline	89.05 ± 10.77	89.19 ± 10.43	0.242
Smoking	298 (31.73)	962 (30.93)	0.357
Hypertension	111 (11.82)	382 (12.28)	0.447
Hyperlipidemia	541 (57.61)	1772 (56.98)	0.479
Diabetes	48 (5.11)	185 (5.95)	0.052

*Continuous variables were expressed as means ± standard deviations when normally distributed, and categorical variables were expressed as percentage of the genotype group (*n* = 4,049).*

### The Relevant Lifestyle and Baseline Characteristics of NAFLD

According to the results shown in Table 4, certain food groups such as milk and beans were associated with a lower risk of NAFLD. Meat, on the other hand, was associated with a higher risk of NAFLD. Fresh fruit and vegetables, salted, and smoked foods, desserts, cereals, fish, and eggs were not associated with the risk of NAFLD. After adjustment for age, this trend remained significant.

**TABLE 4:** Food groups in relation to the risk of NAFLD.

Characteristics	*P*-value	Odds Ratio (95% Confidence Interval)
Fresh fruit and vegetables	<0.001	
Seldom		1 (ref)
1 to 4 times	0.176	0.674 (0.380–1.194)
5 to 7 times	0.005	0.439 (0.249–0.775)
Meat	<0.001	
Seldom		1 (ref)
1 to 4 times	0.001	1.698 (1.226–2.353)
5 to 7 times	<0.001	2.400 (1.731–3.327)
Milk and products	<0.001	
Seldom		1 (ref)
1 to 4 times	<0.001	0.646 (0.555–0.752)
5 to 7 times	<0.001	0.548 (0.427–0.703)
Bean products	<0.001	
Seldom		1 (ref)
1 to 4 times	<0.001	0.710 (0.607–0.831)
5 to 7 times	<0.001	0.701 (0.576–0.853)
Salted and smoked foods	0.005	
Seldom		1 (ref)
1 to 4 times	0.215*	
5 to 7 times	0.014*	
Desserts	<0.001	
Seldom		1 (ref)
1 to 4 times	<0.001	1.371 (1.171–1.605)
5 to 7 times	0.302	1.181 (0.861–1.621)
Cereals	0.103	
Seldom		1 (ref)
1 to 4 times	0.033*	
5 to 7 times	0.043*	
Fish	0.148	
Seldom		1 (ref)
1 to 4 times	0.355*	
5 to 7 times	0.538*	
Eggs	0.623	
Seldom		1 (ref)
1 to 4 times	0.764*	
5 to 7 times	0.472*	

Food intake was divided into three categories, seldom eat, 1 to 4 times a week, 5 to 7 times a week with seldom eat as the reference category. Meat contains Pork, beef, mutton, and poultry. Bean products contain soy bean milk, tofu, and other bean products. Milk and products contain milk and yogurt. Fish contains fish, seafood, and shellfish. Cereals contain rice, pasta, breads, and noodles, (*n* = 4,049). *These factors were not included in the regression equation, and there is no OR value.

### Comparison of the Effects of Food Groups Between the C- and the T-Allele Carriers

Table 5 shows comparison of risk factors of NAFLD between the C- and T-allele carriers. The influence of most food groups, such as desserts, fresh fruit, and vegetables, meat, cereals, fish, and eggs was not significantly different between CC and TT + CT genotype groups. However, it was marginally different for other food groups, namely milk and beans. In addition, the influence of salted and smoked foods on NAFLD was obviously different between the two groups. The intake of salted and smoked foods was found to be a risk factor for NAFLD in the TT and CT groups (OR = 1.196, 95% CI: 1.007–1.420, *P* = 0.041 for 1–4 times food per week intake. OR = 1.580, 95% CI: 1.156–2.159, *P* = 0.004 for 5–7 times per week intake), but it had no effect on the CC genotype group. According to the results shown in Table 5, ORs and 95% CIs had the same sense of base characteristics in relation to the risk of NAFLD. In addition, regular consumption (i.e., several times/week) of salted and smoked foods was associated with a higher risk of developing NAFLD than occasional consumption. After adjustment for age, this trend with salted and smoked foods intake remained significant.

**TABLE 5:** Comparison of the relationship between food groups and NAFLD in the C- and T- allele carriers.

	CC genotype		CT + TT genotype	
	*P*-Value	Odds Ratio (95% Confidence Interval)	*P*-Value	Odds Ratio (95% Confidence Interval)
Milk and products	0.008		<0.001	
Seldom		1 (ref)		1 (ref)
1 to 4 times a week	0.063	0.753 (0.559–1.015)	<0.001	0.628 (0.527–0.747)
5 to 7 times a week	0.003	0.508 (0.323–0.798)	<0.001	0.589 (0.441–0.787)
Bean products	0.011		0.001	
Seldom		1 (ref)		
1 to 4 times a week	0.003	0.612 (0.443–0.847)	0.001	0.745 (0.622–0.892)
5 to 7 times a week	0.293	0.807 (0.541–1.204)	<0.001	0.670 (0.535–0.839)
Desserts	0.460		<0.001	
Seldom		1 (ref)		1 (ref)
1 to 4 times a week	0.250*		<0.001	1.436 (1.198–1.721)
5 to 7 times a week	0.912*		0.288	1.218 (0.847–1.750)
Fresh fruit and vegetables	0.004		<0.001	
Seldom		1 (ref)		1 (ref)
1 to 4 times a week	0.954	0.969 (0.327–2.872)	0.091	0.546 (0.271–1.101)
5 to 7 times a week	0.320	0.580 (0.198–1.697)	0.004	0.361 (0.179–0.725)
Meat	0.002		<0.001	
Seldom		1 (ref)		1 (ref)
1 to 4 times a week	0.002	2.938 (1.463–5.903)	0.047	1.455 (1.005–2.108)
5 to 7 times a week	<0.001	3.512 (1.751–7.046)	<0.001	2.197 (1.516–3.185)
Salted and smoked foods	0.286		0.008	
seldom		1 (ref)		1 (ref)
1 to 4 times a week	0.331*		0.041	1.196 (1.007–1.420)
5 to 7 times a week	0.372*		0.004	1.580 (1.156–2.159)
Cereals	0.390		0.311	
Seldom		1 (ref)		1 (ref)
1 to 4 times a week	0.173*		0.129*	
5 to 7 times a week	0.210*		0.132*	
Fish	0.243		0.248	
Seldom		1 (ref)		1 (ref)
1 to 4 times a week	0.502*		0.154*	
5 to 7 times a week	0.109*		0.717*	
Eggs	0.480		0.436	
Seldom		1 (ref)		1 (ref)
1 to 4 times a week	0.322*		0.800*	
5 to 7 times a week	0.592*		0.679*	

Data were obtained from logistic regression analysis based on Chinese subjects with complete co variable data who were control group or NAFLD group in Health Management Institute (*n* = 4,049). *These factors were not included in the regression equation, and there is no OR value.

### Association Between *MTHFR* Genotype and Blood Biochemical Indicators

Table 6 shows the results of blood lipids, blood glucose, hepatic function, hemoglobin, and homocysteine in the NAFLD and control groups between the C- and the T- allele carriers. Levels of ALT, AST, TC, TG, Hb, Glu, and Hcy of the CC genotype groups were significantly different between the NAFLD and control groups. On the contrary, no significant association was found in HDL-C in CC genotype between the NAFLD and control groups. Levels of ALT, AST, TG, Hb, and Hcy were significantly differences between the CT and TT genotype groups. However, no significant differences were observed for TC, HDL-C, and Glu between the NAFLD and control groups. Homocysteine content was higher in the NAFLD group than in the control group, and also higher in the CT + TT genotype group than in the CC group. The homocysteine content was the highest in the CT + TT genotype group (Table 6).

**TABLE 6:** Comparison of the levels of blood lipids, blood glucose, hepatic markers, hemoglobin, and homocysteine between the C- and T-allele carriers.

	CC genotype groups			CT and TT genotype groups		
	NAFLD	Control	*P*-value	NAFLD	Control	*P*-value
ALT	28.21 ± 20.26	18.47 ± 12.44	<0.001	27.89 ± 17.16	19.11 ± 20.34	<0.001
AST	22.19 ± 11.75	19.33 ± 7.69	<0.001	21.76 ± 11.62	20.02 ± 22.60	0.005
TC	4.83 ± 0.81	4.66 ± 0.68	0.001	4.89 ± 0.81	4.82 ± 0.79	0.22
TG	2.43 ± 1.95	1.91 ± 2.27	<0.001	2.47 ± 1.89	1.79 ± 1.47	<0.001
HDL-C	1.47 ± 0.91	1.55 ± 0.87	0.189	1.44 ± 0.91	1.44 ± 0.64	0.802
Hb	151.13 ± 12.64	140.97 ± 16.08	<0.001	150.23 ± 13.33	139.73 ± 15.68	<0.001
Glu	5.31 ± 1.51	4.82 ± 0.58	<0.001	5.92 ± 1.27	5.46 ± 0.79	0.095
Hcy	11.79 ± 3.51	9.84 ± 3.28	<0.001	14.61 ± 5.89	13.24 ± 5.63	<0.001

*Continuous variables were expressed as means ± standard deviations when normally distributed. TC, total cholesterol, mmol/L; TG, triglycerides, mmol/L; HDL-C, high-density lipoprotein cholesterol, mmol/L; Glu, fasting blood glucose, mmol/L; ALT, alanine aminotransferase, U/L; AST, aspartate aminotransferase, U/L; Hb, hemoglobin, g/L; Hcy, homocysteine, μmol/L.*

### Comparison of the Effects of the Salted and Smoked Foods on Liver Fibrosis

According to the BARD score, there were 528 patients with liver fibrosis in the CC group and 1,838 in the CT + TT group. The influence of salted and smoked foods on liver fibrosis was significantly different between the two groups (Table 7). The intake of salted and smoked foods was found to be a risk factor for the development of liver fibrosis in the TT and CT genotype group (OR = 1.429, 95% CI: 1.040–1.962, *P* = 0.028 for 1–4 times food per week intake. OR = 2.231, 95% CI: 1.170–4.256, *P* = 0.015 for 5–7 times food per week intake), but there was no comparable effect in the CC genotype group (Table 7). Additionally, regular consumption (i.e., several times/week) of salted and smoked foods was associated with a higher risk of developing liver fibrosis than occasional consumption.

**TABLE 7:** Comparison of the relationship between salted and smoked foods and liver fibrosis in the C- and T-allele carriers.

	CC genotype		CT + TT genotype	
	*P*-Value	Odds Ratio (95% Confidence Interval)	*P*-Value	Odds Ratio (95% Confidence Interval)
Salted and smoked foods	0.685		0.013	
Seldom				1 (ref)
1 to 4 times a week	0.496		0.028	1.429 (1.040–1.962)
5 to 7 times a week	0.759		0.015	2.231 (1.170–4.256)

*Data were obtained from logistic regression analysis based on Chinese subjects with complete co-variable data who were NAFLD group in Health Management Institute (*n* = 2,602).*

## Discussion

### Risk Factors of NAFLD and *MTHFR* Genotypes

Our results indicate a relationship between lifestyle and NAFLD in different *MTHFR* genotype groups. First, the occurrence of NAFLD was significantly higher in participants with the CT or TT genotype than in those with the CC genotype. This indicated that the TT genotype of *MTHFR* was more likely to be related to susceptibility to NAFLD (Serin et al., 2007; Liew and Gupta, 2015). *MTHFR* variation was significantly associated with NAFLD risk in a Turkish study (Sazci et al., 2008), and our results were consistent with that study. Second, our results indicated that the risk factors of NAFLD are age, sex, BMI, smoking, hypertension, and diabetes, which was consistent with another review of literature (Benedict and Zhang, 2017). Lonardo et al’s epidemiological review (Non-alcoholic Fatty Liver Disease Study Group, Lonardo et al., 2015) showed NAFLD was more common in men. Similarly, our study found that the prevalence of NAFLD in males was 82.59%. Third, in the CC-genotype group, the levels of ALT, AST, TG, Hb, and Hcy were obviously different in the NAFLD and control groups, and the results were the same in the CT- and TT-genotype groups. We concluded that these changes were related to NAFLD. Kwo et al. (2017) reported that the degree of elevation of ALT and or AST levels could aid in the assessment of NAFLD. Similarly, Cohen et al., reported that patients with NAFLD exhibited an increased plasma concentration of TG and TC (Cohen and Fisher, 2013). Moreover, Hu et al. (2019) suggested that Glu levels are related to NAFLD. Hb has also been found to be associated with NAFLD (Jiang et al., 2014). The association of the *C677T* gene variant of *MTHFR* with total cholesterol were consistently more marked in Asian populations (Luo et al., 2018). Luo et al. (2018) found that the *C677T* gene variant was associated with increased risk of coronary artery disease and elevated levels of total cholesterol. However, we found that total cholesterol levels were different between the C- and T-allele carriers. The mechanisms by which *MTHFR* variation is associated with TC levels have not been clarified yet. Homocysteine levels were also reported to be associated with NAFLD (Liang et al., 2019).

### Influence of Food Groups on *MTHFR* Genotypes

We found that certain food groups such as milk and beans were associated with a lower risk of NAFLD, whereas meats were associated with a higher risk of NAFLD. According to the results of the logistic regression, the influence of most food groups on NAFLD was not significantly different between the CC and TT + CT groups. However, the influence of salted and smoked foods was obviously different between the two groups. After adjustment for age, this trend of salted and smoked foods intake remained significant. We speculate that this phenomenon is likely related to the choline content in the food. Importantly, choline has been found to be associated with *MTHFR* variation, and Jian Yan et al.’s findings indicate that the *MTHFR C677T* genotype favors the use of choline as a methyl donor (Yan et al., 2011). The liver is an important organ for metabolism and storage of choline (Zeisel, 2006). Studies have shown that choline deficiency can cause NAFLD and non-alcoholic steatohepatitis (NASH) (Yao and Vance, 1988; Corbin and Zeisel, 2012; Matsumoto et al., 2013). The *MTHFR C677T* variation can lead to hyperhomocysteinemia (Liew and Gupta, 2015), and the lack of choline can lead to hyperhomocysteinemia (Liu et al., 2014). Schwahn et al. reported that betaine supplementation of *MTHFR*-deficient mice reduced lipid deposition in the liver, and increased homocysteine and choline metabolism (Schwahn et al., 2003). We found that the content of homocysteine was higher in the NAFLD group than in the control group, and the homocysteine content was the highest in the T-genotype group of NAFLD. Therefore, we speculate that choline is deficient in the T-genotype group of NAFLD. In addition, foods that contain choline include meat, fruits and vegetables, milk, beans, desserts, and salted and smoked foods, but the choline content is lower in salted and smoked foods. For example, Frankfurter sausages have less choline than unprocessed meat (Probst et al., 2019). Therefore, adults who consumed salted and smoked foods were more susceptible to NAFLD. We also found that salted and smoked foods were a risk factor for NAFLD in the T-genotype group, while this effect was not seen in the C-genotype group. The likely reason may be that choline content is lower in salted and smoked foods, which is more strongly associated with NAFLD. Similarly, salted and smoked foods intake was a risk factor for liver fibrosis in the TT + CT group in patients with NAFLD. Importantly, patients with NAFLD who consumed salted and smoked foods were more susceptible to NASH. These results indicate that food intervention may be more effective for reducing the prevalence of NAFLD in different genotype groups.

### Limitations

Our study has some limitations. First, we combined the CT and TT genotypes as one group. The *C677T* mutation represents the substitution of C (cytosine) for T (thymine) at nucleotide position 677, which results in the reduction of MTHFR enzyme activity. Therefore, including the T genotype as a group may have affected the MTHFR enzyme activity. Second, we only studied choline in certain foods, and intend to study more food groups in the future. Third, although NAFLD is a multifactorial disease, we only studied the relationship between diet and NAFLD. Moreover, we only studied the homocysteine levels and lack nutrient status/biochemical data (e.g., choline plasma levels). We plan to study the other biochemical data in future studies. Finally, we only studied a limited range of consumption quantities of each food, and aim to further evaluate the daily consumption quantities of each food by individuals in future studies.

### Conclusion

This study’s findings indicated that *MTHFR* CT and TT genotypes are risk factors for NAFLD among Chinese people. The results also suggested that the genetic risk factor may interact with dietary factors, particularly salted and smoked foods. The interaction between food groups intake and *MTHFR C677T* variation may affect NAFLD incidence in the adult Chinese population.

## Data Availability Statement

The original contributions presented in the study are included in the article/supplementary material, further inquiries can be directed to the corresponding author.

## Ethics Statement

The studies involving human participants were reviewed and approved by the Institutional Ethics Committee of Chinese PLA General Hospital. The patients/participants provided their written informed consent to participate in this study. Written informed consent was obtained from the individual(s) for the publication of any potentially identifiable images or data included in this article.

## Author Contributions

XH, CM, TX, LO, and QZ: data curation. XH: investigation. CM and QZ: methodology. TX and LO: software. XH and CM: writing–original draft. XH and QZ: writing–review and editing. All authors contributed to the article and approved the submitted version.

## Conflict of Interest

The authors declare that the research was conducted in the absence of any commercial or financial relationships that could be construed as a potential conflict of interest.
